# Supplementary material for: Expression of Cellular and Extracellular TERRA, TERC and TERT in Hepatocellular Carcinoma
Source: Int J Mol Sci. 2022 May 31;23(11):6183. doi: 10.3390/ijms23116183 (PMC9181112; doi:10.3390/ijms23116183)
Supplement: Supplementary file 1 [file ijms-23-06183-s001.zip › ijms-1724131-supplementary/Supplementary Figures/Figure S1.pdf]

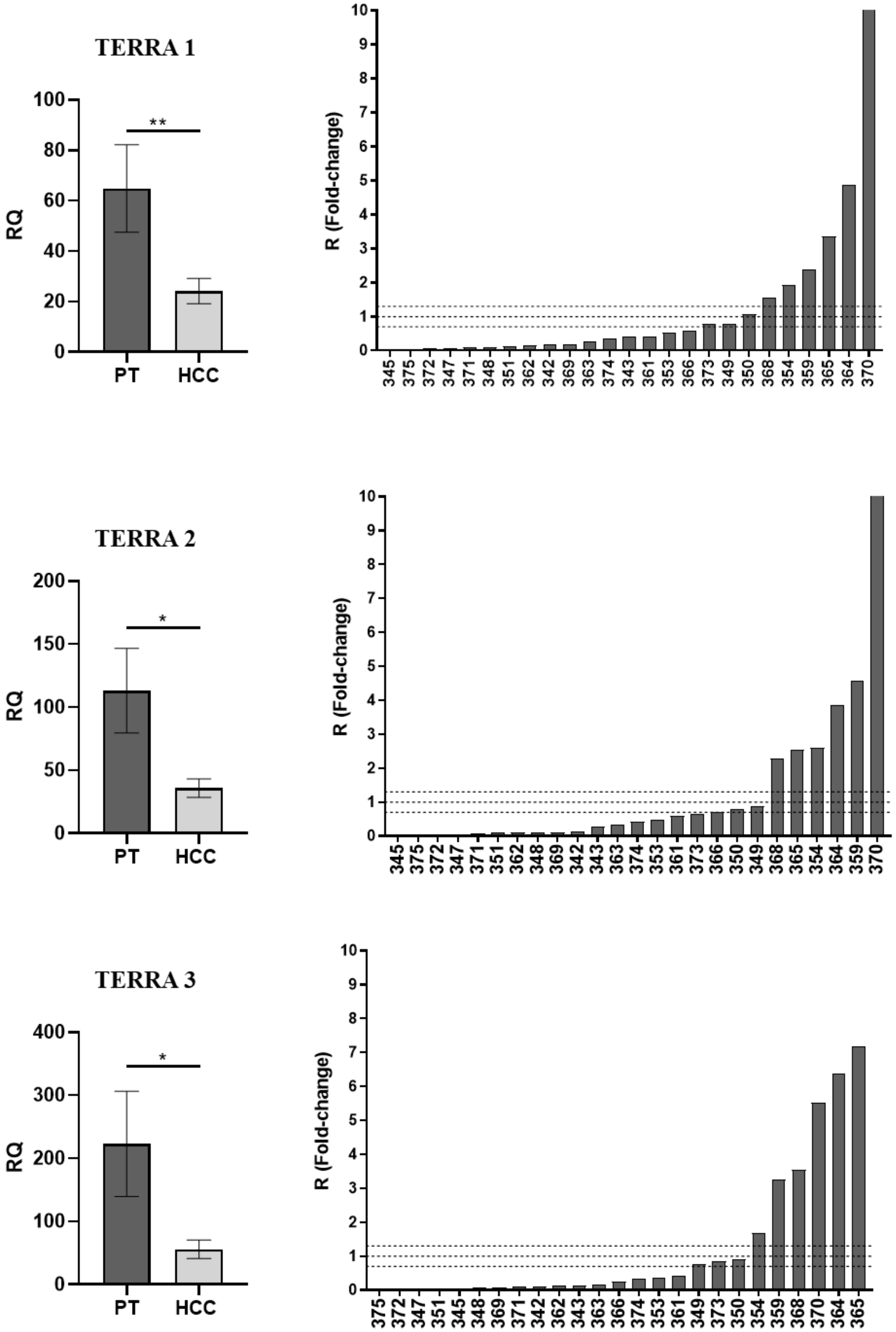

Figure S1. Tissue expression levels of TERRA 1, TERRA 2, and TERRA 3. On the left, relative quantification mean comparison between PT and HCC for each TERRA locus. Wilcoxon test was used; *$p<0.05$, **$p<0.01$. On the right, relative TERRA Fold-change. Histograms represent R-values. Histograms are ordered by increasing R-values. The corresponding cases are listed on x axis. Upper and lower dot lines define the range of expression variation.
