# Supplementary material for: Expression of Cellular and Extracellular TERRA, TERC and TERT in Hepatocellular Carcinoma
Source: Int J Mol Sci. 2022 May 31;23(11):6183. doi: 10.3390/ijms23116183 (PMC9181112; doi:10.3390/ijms23116183)
Supplement: Supplementary file 1 [file ijms-23-06183-s001.zip › ijms-1724131-supplementary/Supplementary Figures/Figure S2.pdf]

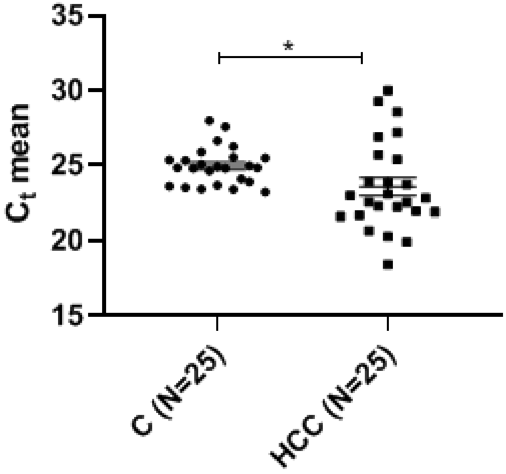

Figure S2. Plasma levels of TERRA. TERRA levels were reported as mean of Ct derived from the analysis of different TERRA targets (TERRA1, TERRA2, and TERRA3) in healthy (C) and HCC individuals. Unpaired t-test was used; *$p<0.05$.
