# Supplementary material for: Expression of Cellular and Extracellular TERRA, TERC and TERT in Hepatocellular Carcinoma
Source: Int J Mol Sci. 2022 May 31;23(11):6183. doi: 10.3390/ijms23116183 (PMC9181112; doi:10.3390/ijms23116183)
Supplement: Supplementary file 1 [file ijms-23-06183-s001.zip › ijms-1724131-supplementary/Supplementary Figures/Figure S3.pdf]

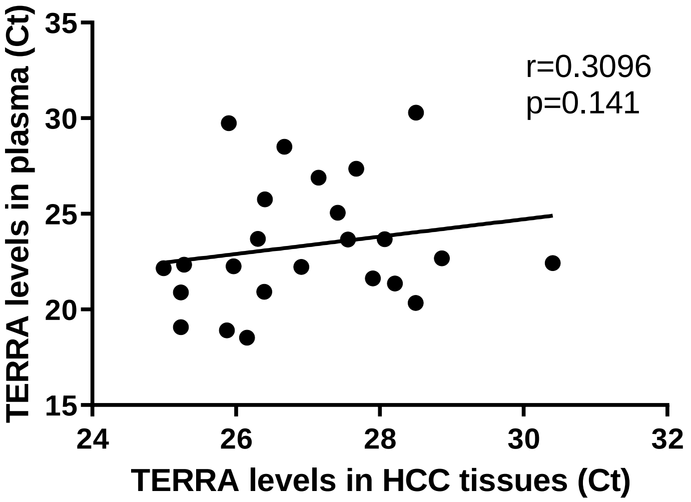

Figure S3. Correlation between the average Ct values for TERRA levels in plasma and in HCC tissues. Correlation was tested with Spearman's correlation analysis.
