# Supplementary material for: Expression of Cellular and Extracellular TERRA, TERC and TERT in Hepatocellular Carcinoma
Source: Int J Mol Sci. 2022 May 31;23(11):6183. doi: 10.3390/ijms23116183 (PMC9181112; doi:10.3390/ijms23116183)
Supplement: Supplementary file 1 [file ijms-23-06183-s001.zip › ijms-1724131-supplementary/Supplementary Figures/Figure S4.pdf]

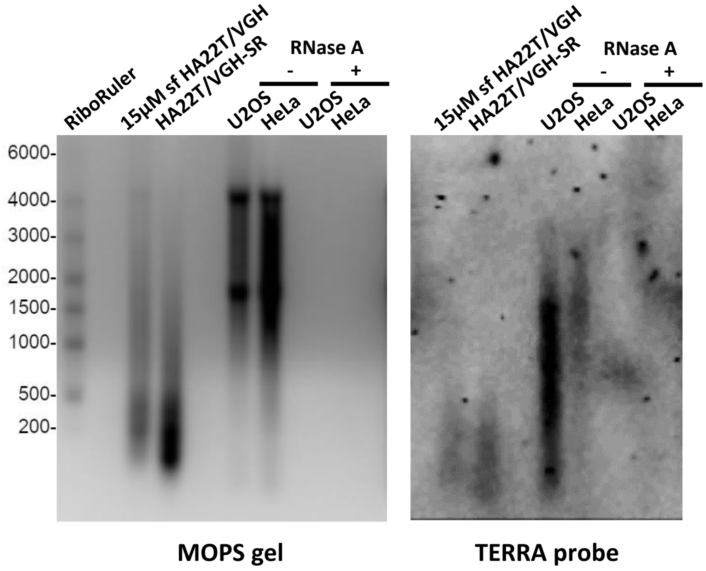

Figure S4. Northern blot analysis of TERRA in EVs from HCC cells. EVs samples from HCC cells contain TERRA in the size range <500 nt. For the Northern blotting, both EVs-contained RNA from HA22T/VGH cells and control total RNA samples were loaded on a gel, with control samples from HeLa and U2OS cell lines, blotted and hybridized with a TERRA-complementary probe. RNase A treatment (30 min. at 37°C) was used as a negative control. The MOPS gel was stained with Xpert Green DNA Stain.
