# Supplementary material for: Expression of Cellular and Extracellular TERRA, TERC and TERT in Hepatocellular Carcinoma
Source: Int J Mol Sci. 2022 May 31;23(11):6183. doi: 10.3390/ijms23116183 (PMC9181112; doi:10.3390/ijms23116183)
Supplement: Supplementary file 1 [file ijms-23-06183-s001.zip › ijms-1724131-supplementary/Table S1.pdf]

Table S1. Association between the clinicopathological characteristics of HCC patients and the TERRA expression levels

	Number of patients	Low expression R < 0.7	Equal/High expression R > 0.7	P-value
**Gender**				
Male	20	12	8	0. 620
Female	5	4	1	
**Age**[#]				
≤ 73 years	13	9	4	0.688
> 73 years	12	7	5	
**Grading**				
G1 – G2	16	11	5	0.671
G2/G3-G3	9	5	4	
**Hepatitis B infection**				
Positive	3	2	1	1.000
Negative	22	14	8	
**Hepatitis C infection**				
Positive	14	10	4	0.434
Negative	11	6	5	
**Cirrhosis**				
Yes	13	11	2	0.082
No	11	5	6	

[#] The median value of years was used as cutoff point.

The associations between the clinicopathological characteristics of HCC patients and the expression levels of TERRA were calculated using Fisher’s exact test.
