# Supplementary material for: Expression of Cellular and Extracellular TERRA, TERC and TERT in Hepatocellular Carcinoma
Source: Int J Mol Sci. 2022 May 31;23(11):6183. doi: 10.3390/ijms23116183 (PMC9181112; doi:10.3390/ijms23116183)
Supplement: Supplementary file 1 [file ijms-23-06183-s001.zip › ijms-1724131-supplementary/Table S2.pdf]

Table S2. Association between the clinicopathological characteristics of HCC patients and the circulating levels of TERRA in HCC patients

	**Number of patients**	**High TERRA levels** Ct ≤ 22.81	**Low TERRA levels** Ct > 22.81	**P-value**
**Gender**				
Male	21	10	11	0.593
Female	4	3	1	
**Age**[#]				
≤ 73 years	12	7	5	0.434
> 73 years	13	5	8	
**Grading**				
G1 – G2	16	9	7	0.688
G2/G3-G3	9	4	5	
**Hepatitis B infection**				
Positive	3	1	2	0.593
Negative	22	12	10	
**Hepatitis C infection**				
Positive	14	8	6	0.999
Negative	11	6	5	
**Cirrhosis**				
Yes	13	7	6	0.999
No	11	6	5	

[#] The median value of years was used as cutoff point.

The associations between the clinicopathological characteristics of HCC patients and the circulating levels of TERRA were calculated using Fisher’s exact test.
