# Supplementary material for: Expression of Cellular and Extracellular TERRA, TERC and TERT in Hepatocellular Carcinoma
Source: Int J Mol Sci. 2022 May 31;23(11):6183. doi: 10.3390/ijms23116183 (PMC9181112; doi:10.3390/ijms23116183)
Supplement: Supplementary file 1 [file ijms-23-06183-s001.zip › ijms-1724131-supplementary/Table S3.pdf]

Table S3. Clinical characteristics of HCC patients enrolled in the study.

Case	Gender	Years	Grading	Background disease	HBV	HCV	AFP ng/ml
342	M	75	G2-G3	Steatosis	-	+	4
343	M	64	G2	Chronic hepatitis	-	+	1
345	M	72	G2	Chronic hepatitis	-	+	-
347	M	63	G2	Active cirrhosis	-	-	8
348	M	60	G3	Chronic hepatitis	+	-	10
349	M	71	G2	Hepatitis	-	-	-
350	M	78	G3	NA	-	+	3
351	F	69	G2	Active cirrhosis	-	+	-
352[#]	M	82	G2	Early cirrhosis	-	+	-
353	M	82	G2	Normal	-	-	763
354	M	71	G1	Steatosis	-	-	-
359	M	75	G2	Cirrhosis	-	+	-
361	M	74	G2	Early cirrhosis with moderate steatosis	-	+	-
362	F	79	G2-G3	Cirrhosis with steatosis	-	+	13
363	M	74	G2	Cirrhosis with steatosis	-	-	-
364	F	77	G3	Cirrhosis with steatosis	-	+	7
365	M	76	G3	Von meyenburg complex	-	-	NA
366	M	65	G2-G3	Cirrhosis with steatosis	+	-	-
368	M	73	G3	Chronic hepatitis	+	-	7
369	M	68	G3	Cirrhosis with steatosis	-	-	-
370	M	74	G2	Chronic hepatitis	-	+	-
371*	F	77	G2	Cirrhosis with steatosis	-	+	2
372	M	73	G2	Early cirrhosis with moderate steatosis	-	+	9
373	M	73	G2	Steatosis	-	-	2
374	F	78	G1	Cirrhosis	-	+	1
375	M	71	G2	Cirrhosis with steatosis	-	+	-

[#] Peripheral blood only; * Solid biopsies only; NA, not applicable.
